# Stimulatory Effects of Balanced Deep Sea Water on Mitochondrial Biogenesis and Function

**DOI:** 10.1371/journal.pone.0129972

**Published:** 2015-06-12

**Authors:** Byung Geun Ha, Jung-Eun Park, Hyun-Jung Cho, Yun Hee Shon

**Affiliations:** Bio-Medical Research Institute, Kyungpook National University Hospital, Daegu, Korea; Universidad Pablo de Olavide, Centro Andaluz de Biología del Desarrollo-CSIC, SPAIN

## Abstract

The worldwide prevalence of metabolic diseases, including obesity and diabetes, is increasing. Mitochondrial dysfunction is recognized as a core feature of these diseases. Emerging evidence also suggests that defects in mitochondrial biogenesis, number, morphology, fusion, and fission, contribute to the development and progression of metabolic diseases. Our previous studies revealed that balanced deep-sea water (BDSW) has potential as a treatment for diabetes and obesity. In this study, we aimed to investigate the mechanism by which BDSW regulates diabetes and obesity by studying its effects on mitochondrial metabolism. To determine whether BDSW regulates mitochondrial biogenesis and function, we investigated its effects on mitochondrial DNA (mtDNA) content, mitochondrial enzyme activity, and the expression of transcription factors and mitochondria specific genes, as well as on the phosphorylation of signaling molecules associated with mitochondria biogenesis and its function in C_2_C_12_ myotubes. BDSW increased mitochondrial biogenesis in a time and dose-dependent manner. Quantitative real-time PCR revealed that BDSW enhances gene expression of PGC-1α, NRF1, and TFAM for mitochondrial transcription; MFN1/2 and DRP1 for mitochondrial fusion; OPA1 for mitochondrial fission; TOMM40 and TIMM44 for mitochondrial protein import; CPT-1α and MCAD for fatty acid oxidation; CYTC for oxidative phosphorylation. Upregulation of these genes was validated by increased mitochondria staining, CS activity, CytC oxidase activity, NAD^+^ to NADH ratio, and the phosphorylation of signaling molecules such as AMPK and SIRT1. Moreover, drinking BDSW remarkably improved mtDNA content in the muscles of HFD-induced obese mice. Taken together, these results suggest that the stimulatory effect of BDSW on mitochondrial biogenesis and function may provide further insights into the regulatory mechanism of BDSW-induced anti-diabetic and anti-obesity action.

## Introduction

The decrease of mitochondrial DNA (mtDNA) and mitochondrial dysfunction play an important role in diverse age-associated disorders and metabolic diseases such as type 2 diabetes and obesity [1]. Emerging evidence suggests that impaired mitochondrial metabolism is accompanied by reduced transcription factor activity regulating mitochondrial biogenesis in patients with insulin resistance, type 2 diabetes and obesity [2, 3].

Mitochondrial biogenesis is regulated by a complex network of transcription factors such as peroxisome proliferator-activated receptor co-activator 1 alpha (PGC-1α), the nuclear respiratory factors (NRFs), and mitochondrial transcription factor A (mtTFA) [4]. Furthermore, reduced mtDNA copy number has also been shown in muscle and adipose tissue from diabetic patients [5]. Treatment with thiazolidinedione (TZD), an insulin-sensitizing drug currently used to treat type 2 diabetes, restored the decreased mtDNA content as well as the expression of genes involved in mitochondrial biogenesis and fatty acid oxidation [6, 7]. Therefore, the promotion of mitochondrial biogenesis and function could be a strategy for preventing and treating metabolic diseases, including insulin resistance, obesity, and diabetes.

Deep-sea water (DSW), that is, sea water found at a depth of more than 200 m, is a stable natural resource and is available in infinite supply as compared to other natural products. It contains high levels of essential minerals such as magnesium (Mg), calcium (Ca), and potassium (K), as well as beneficial trace minerals for human health, such as chromium (Cr), selenium (Se), zinc (Zn), and vanadium (V). Several studies investigating the physiological effects of DSW at various degrees of hardness found that DSW might aid in preventing hypertension [8], atopic eczema/dermatitis syndrome [9], and arteriosclerosis [10].

Previously, we demonstrated that balanced DSW (BDSW), which is a mixture of DSW mineral extracts and desalinated water, has anti-diabetic potential via the inhibition of hyperglycemia and improvement in glucose intolerance by increasing glucose uptake in type 1 [11] and 2 diabetic mice [12]. In addition, BDSW has antiobesity potential because it can inhibit adipocyte hypertrophy and reduce liver steatosis in high-fat diet (HFD)–induced obese mice [13]. Reduced mitochondrial content has also been observed in hypertrophic epididymal fat and fatty livers of HFD-induced obese mice, suggesting a potential role for the disruption of mitochondrial content in adipose and liver tissue during development of obesity and type 2 diabetes. Interestingly, drinking BDSW remarkably increased mitochondrial biogenesis by inducing the expression of the main genes involved in its regulation such as PGC-1α, NRF1, and mtTFA in the adipose and liver of HFD-induced obese mice [13]. A decrease in mitochondrial function or mtDNA content has been correlated with the degree of insulin resistance and dysregulated lipid metabolism [14].

Based on the above findings, we hypothesized that the increase in mitochondrial content and expression of transcription factor genes by BDSW could stimulate mitochondrial function and prevent diabetes and obesity-related mitochondrial dysfunction. Therefore, in the present study, we first determined whether BDSW could stimulate mitochondrial function by stimulating mitochondrial biogenesis and its regulatory mechanism in C_2_C_12_ myotubes. To expand on the information obtained in a previous study, we investigated the effect of drinking BDSW on mitochondrial biogenesis in the muscles of HFD-induced obese mice.

## Materials and Methods

### Ethics statement

No permission was required for the areas studied involving collection of water. Animal experiments were performed by the guidelines established by the Animal Ethics Committee of Kyungpook National University, and the protocols were approved by this committee (Approval No. KNU 2012–88).

### Materials

The C_2_C_12_ cell line was purchased from the American Type Culture Collection (ATCC No.CRL-1772, Manassas, VA, USA). The following items were purchased from the stated commercial sources: 5-amino-1-β-d-ribofuranosyl-imidazole-4-carboxamide (AICAR), nicotinamide, ethidium bromide (EtBr), 4′,6-Diamidino-2-phenylindole (DAPI) from Sigma-Aldrich Co. LLC. (St. Louis, MO, USA); anti-phospho AMPKα (Thr172), anti-AMPKα and anti-phospho SIRT1 (Ser47) from Cell Signaling Technology (Danvers, MA, USA); citrate synthase (CS) assay kit, cytochrome oxidase (COX) activity assay kit, and NAD^+^/NADH quantification kit from BioVision, Inc. (Milpitas, CA, USA); SRT1720, protein A/G agarose, anti-SIRT1, anti-PGC-1α, anti-acetylated-lysine (Ac-Lys), anti-β-actin, horseradish peroxidase (HRP)-conjugated anti-mouse IgG, and anti-rabbit IgG-HRP antibodies from Santa Cruz Biotechnology Inc. (Santa Cruz, CA, USA); ECL Plus Western Blotting Substrate from Pierce Biotechnology (Rockford, IL, USA); TRIzol and MitoTracker Red CMXRos from Invitrogen Life Technologies (Carlsbad, CA, USA); PrimeScript 1st strand cDNA Synthesis Kit from Takara Bio Inc. (Shiga, Japan); FastStart Universal SYBR Green Master from Roche Applied Science (Basel, Switzerland); Phosphatase Inhibitor Cocktail and Protease Inhibitor Cocktail solutions from GenDEPOT (Barker, TX, USA).

### BDSW preparation

As described in detail previously [11], original DSW that had been pumped up from a depth of 0.5 km and a distance of 6.7 km off Oho-Ri, Goseong (38°20´ N and 128°34´ E, Gangwon-Do, Korea) was filtered using a microfilter system (Synopex INC, Pohang, Korea) to remove phytoplankton and microorganisms. Next, the filtered DSW was passed through a reverse osmosis membrane (Vontron Technology Co. Ltd., Beijing, China) to obtain DSW mineral extracts and desalinated water. The BDSW was serially diluted using desalinated DSW with regard to hardness. In this study, we defined the hardness of BDSW by using the concentrations of Mg and Ca. The hardness was calculated according to the following equation: Hardness = Mg (mg/L) × 4.1 + Ca (mg/L) × 2.5 [15]. The mineral content of samples were measured by Dionex ICS-1100 basic integrated ion chromatography system (Thermo Scientific Inc., Sunnyvale, CA, USA) for cation analysis such as Ca and Mg, and ELAN 9000/6X00/DRC-e ICP-MS (PerkinElmer Inc., Waltham, MA, USA) for trace minerals analysis such as Se, V, and Zn. S1 Table indicates the mineral content of the original DSW and BDSW samples with a hardness of 4680 ppm.

### Cell culture and myotube differentiation

As described in detail previously [11], murine C_2_C_12_ myoblast was maintained in Dulbecco’s modified Eagle medium (DMEM) containing 10% FBS (HyClone Laboratories, South Logan, UT, USA) and 1% antibiotics (penicillin-streptomycin) in 5% CO_2_ at 37°C. To induce myotubes differentiation, C_2_C_12_ myoblasts were incubated in differentiation medium containing DMEM with 2% horse serum (Life Technologies Inc., Grand Island, NY, USA) for 2 days after reaching confluency. The media was changed daily, and C_2_C_12_ myotubes were prepared for experiments 4–5 days after plating.

### Animal experiment

To determine the effects of BDSW on mitochondrial biogenesis in muscles of HFD-induced obese mice, we used muscles isolated from a previous animal study [13]. Briefly, the C57BL/6J mice (6 weeks of age) in each of the 5 groups (eight mice per group) were given either the normal diet (D12450B, 10% kcal% fat; Research diet, New Brunswick, NJ, USA), HFD (D12451, 45% kcal% fat; Research diet, New Brunswick, NJ, USA), or HFD with BDSW of differing hardness (500, 1000, and 2000 ppm) for 20 weeks. At the end of the feeding period, muscles was collected under anesthesia with zoletil (Virbac S.A, Carros, France). All samples were stored at −70°C until assayed.

### Mitochondrial biogenesis analysis

To assess mitochondrial biogenesis, C_2_C_12_ myotubes were incubated in DMEM containing desalinated water, BDSW at different levels of hardness (0, 100, 500, 1000, 1500, 2000), or 1 mM AICAR (AC) for the indicated time (0, 1, 2, 3 days). To investigate the effect of inhibitors on mitochondrial biogenesis, C_2_C_12_ myotubes were incubated with BDSW at hardness 2000 only and BDSW 2000 ppm with inhibitors such as 2 mM nicotinamide (NA), 10 μM compound C (CC), and 20 ng/ml EtBr, and 1 mM AICAR (AC) for 1 day. As described in detail previously [13], mitochondrial biogenesis was determined by the ratio of mitochondrial DNA (mtDNA) to nuclear DNA (nDNA), quantified by real-time qPCR, assuming that nDNA levels remain constant. DNA was extracted from C_2_C_12_ myotubes and muscles of HFD-induced obese mice, using a genomic DNA purification kit (Promega Corp., Madison, WI, USA), according to the manufacturer’s instructions. For each DNA extract, mRNA levels of the nuclear gene ribosomal protein large p0 (RPLP0) and the mitochondrial gene cytochrome c oxidase subunit I (COX I) were quantified by quantitative real-time polymerase chain reaction (qRT-PCR). All primers are listed in S2 Table. SYBR Green was used to measure expression levels and data were normalized against the expression of RPLP0 (ΔΔCT analysis).

### Mitochondrial staining and quantification

C_2_C_12_ myotubes were differentiated in 8-well chamber slides (Thermo Scientific Inc., Rochester, NY, USA) and then treated with DMEM containing BDSW 2000, 1 mM AICAR, or 10 μM SRT1720 for 24 h. Next, myotubes were incubated with DMEM containing 20 nM Mito Tracker Red CMXRos at 37°C for 30 min. After fixing with 4% paraformaldehyde, cells were stained with 1 μg/mL DAPI for 10 min. Image acquisition was performed using a digital microscope (Nikon eclipse 80i; Tokyo, Japan) at × 400 magnification. As described in detail previously [11], to quantify the fluorescent signal, cells were washed with PBS and incubated with lysis buffer (0.1 M potassium phosphate, 1% Triton X-100, pH 10) for 10 min with shaking. Subsequently, DMSO was added and the cells were allowed to shake for 10 min. The intensity of the fluorescent signal was measured using a microplate reader (FLUOstar OPTIMA, BMG LABTECH, Germany) at excitation and emission wavelengths of 466 nm and 540 nm, respectively.

### Citrate synthase activity, cytochrome c oxidase activity, and NAD^+^/NADH ratio analysis

After C_2_C_12_ myotubes were incubated with DMEM containing BDSW at different hardness (0, 100, 500, 1000, 1500, 2000 ppm), or 1 mM AICAR for 6 h, enzyme activity was measured using citrate synthase activity kit and cytochrome c oxidase activity kit (BioVision, Inc. Milpitas, CA, USA), and the NAD^+^ and NADH content was determined using NAD^+^ /NADH quantification kit (BioVision, Inc. Milpitas, CA, USA) in cell lysates (10 μg), respectively, according to the manufacturer's instructions. Using a microplate reader (Model AD200; Beckman Coulter, Brea, CA, USA), citrate synthase (CS) and cytochrome c oxidase (COX) activity were determined using a kinetic program at OD 412 nm and OD 550 nm, respectively. The amount of total NAD^+^ or NADH in the lysates was determined at OD 450 nm. The ratio of NAD^+^ to NADH was calculated by dividing the total NAD^+^ or NADH concentration by the total protein concentration.

### Nuclear extraction and immunoprecipitation to assess PGC-1α activity

To assess PGC-1α activity, C_2_C_12_ myotubes were incubated with DMEM containing BDSW at different hardness levels, 1 mM AICAR, or 10 μM SRT1720 for 6 h. As described in detail previously [16], immunoprecipitation of PGC-1α was performed using the Nuclear Complex Co-IP Kit (Active Motif Inc. Carlsbad, CA, USA), according to manufacturer’s instructions. Briefly, PGC-1α was immunoprecipitated from nuclear extracts (200 μg) using a rabbit anti-PGC-1α polyclonal antibody (Santa Cruz, CA, USA) with gentle rocking overnight at 4°C and was then incubated with protein A/G plus agarose beads (40 μl of 50% bead slurry; Santa Cruz, CA, USA) for 2 h at 4°C. Subsequently, immunoprecipitates were immunoblotted with either an anti-acetylated lysine antibody to determine the extent of PGC-1α acetylation (Ac-Lys) or a rabbit anti-PGC-1α polyclonal antibody to determine the total amount of PGC-1α.

### Western blot analysis

For determination of AMPK and SIRT1 activity, C_2_C_12_ myotubes were incubated with DMEM containing BDSW with differing hardness (0, 100, 500, 1000, 1500, 2000 ppm), 1 mM AICAR, or 10 μM SRT1720 for 1 h, and then the levels of AMPK and SIRT1 phosphorylation were analyzed. To investigate the effect of several inhibitors on AMPK and SIRT1 phosphorylation, the cells were preincubated in DMEM in the presence or absence of 2 mM nicotinamide (NA), 10 μM compound C (CC), and 20 ng/ml EtBr for 1 h, and then the cells were incubated in DMEM containing BDSW (hardness, 2000 ppm) only, with or without inhibitors for 1 h. After incubation was completed, the cells were washed with PBS, and lysed in RIPA buffer (50 mM NaCl, 10 mM Tris, 0.1% SDS, 1% Triton X-100, 0.1% sodium deoxycholate, 5 mM EDTA, 1 mM Na_3_VO_4_, pH 7.4). Equal amounts of cell lysate protein (40 μg/lane) was subjected to SDS-polyacrylamide gel electrophoresis and transferred to a nitrocellulose membrane (Whatman, Dassel, Germany). The membrane was blocked with 5% non-fat dried milk (BD Bioscience, San Jose, CA) for 1 h and incubated with anti-PGC-1α, anti-phospho AMPKα (Thr172), anti-AMPK, anti-phospho SIRT1 (Ser47), anti-SIRT1, anti-acetylated-lysine (Ac-Lys), and anti-β-actin (diluted 1:1000) overnight at 4°C. After washing with TBS containing 0.1% Tween-20, the membrane was incubated with HRP-conjugated anti-mouse IgG or anti-rabbit IgG antibodies (diluted 1:3000) for 1 h at room temperature. Antibody binding on the nitrocellulose membrane was detected with an ECL plus solution and radiography. The images were detected with a lumino image analyzer (Model LAS-4000 Mini; Fujifilm, Tokyo, Japan) and coupled with image analysis software (Multi Gauge Ver. 3.0; Fujifilm).

### Quantitative real time- polymerase chain reaction (qRT-PCR) analysis

As described in detail previously [11], total RNA was isolated from C_2_C_12_ myotubes and muscles of HFD-induced obese mice using TRIzol, and cDNA was synthesized using PrimeScript1st strand cDNA synthesis kit (Takara Bio Inc., Shiga, Japan), according to the manufacturer’s instructions. qRT-PCR was performed in triplicate using a FastStart SYBR Green Master Mix in an ABI Prism 7300 Sequence Detection System (Applied Biosystems, Foster City, CA, USA). The expression levels of target genes relative to that of the endogenous reference gene actin were calculated using the delta cycle threshold method. The primer sequences are listed in S2 Table.

### Statistical analysis

All results were compared by one-way analysis of variance (ANOVA) using Statistical Package for the Social Sciences (SPSS, ver. 11.0), and are expressed as means ± SE in three independent experiments. Group means were considered as significantly different at *p* < 0.05, as determined by the technique of protective least significant difference when ANOVA indicated an overall significant treatment effect of *p* < 0.05.

## Results

### BDSW enhances mitochondrial biogenesis in C_2_C_12_ myotubes

**Fig 1:**
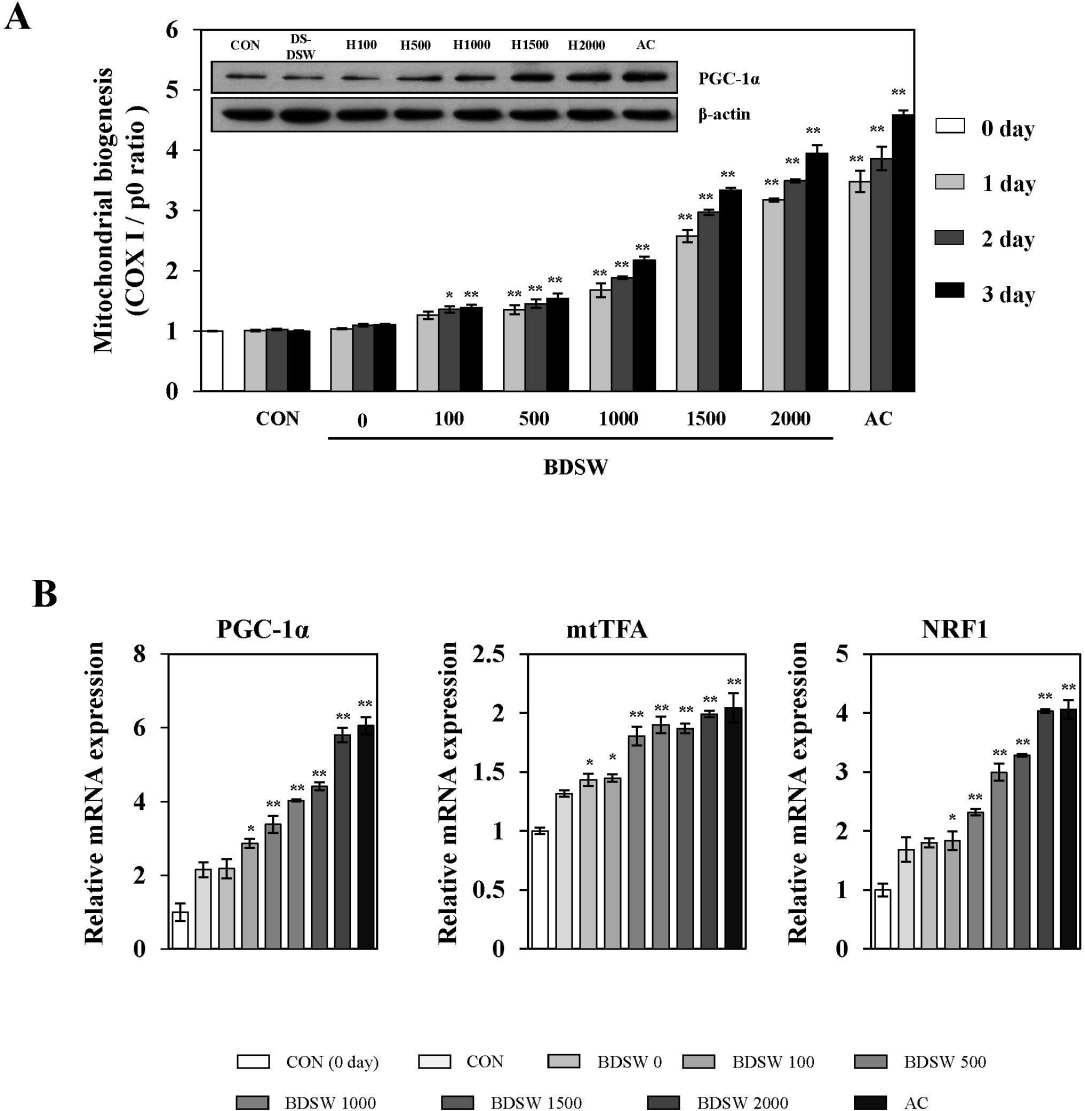
Effect of BDSW on mtDNA, expression of PGC-1α (A), and PGC-1α, mtTFA, and NRF1 gene expression (B) in C_2_C_12_ myotubes. Myotubes were incubated in DMEM containing desalinated water, BDSW at different levels of hardness (0, 100, 500, 1000, 1500, 2000), or 1 mM AICAR (AC) for the indicated time (0, 1, 2, 3 days). All media were changed daily. Expression of PGC-1α was determined by western blot analysis (3 days). β–actin served as the standard. B. After C_**2**_C_**12**_ myotubes were incubated with BDSW at different levels of hardness for 24 h, gene expression of markers of mitochondrial biogenesis were determined by quantitative real-time PCR. Each value represents the mean ± SE of three independent experiments. *P < 0.05, **P < 0.01: significant difference vs. 0 day (A) or CON group (B). CON group: DMEM media treated control group; DS-W, desalinated water.

**Fig 2:**
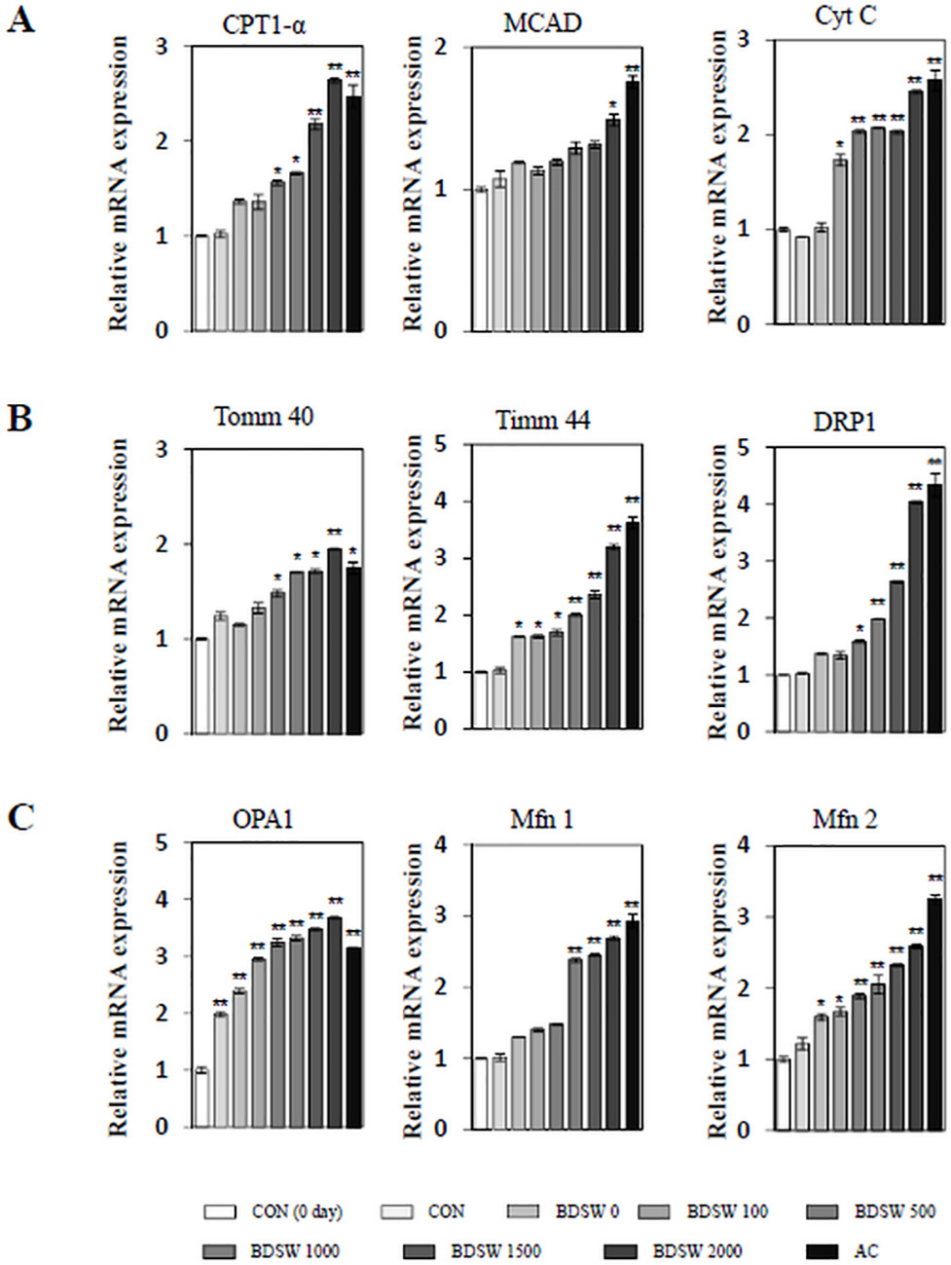
Effect of BDSW on expression of genes involved in fatty acid oxidation, oxidative phosphorylation, and mitochondrial dynamic in C_2_C_12_ myotubes. After C_**2**_C_**12**_ myotubes were incubated in DMEM containing desalinated water, BDSW at different levels of hardness or 1 mM AICAR (AC) for 24 h, expression of CPT1-α and MCAD for fatty acid oxidation and CytC for oxidative phosphorylation (A), TOMM 40 and TIMM 44 for mitochondrial protein import and DRP1 for mitochondrial fission (B), and OPA1, MFN1, and MFN2 for mitochondrial fusion (C) were determined by quantitative real-time PCR. Each value represents the mean ± SE of three independent experiments. *P < 0.05, **P < 0.01: significant difference vs. CON group. CON group: DMEM media treated control group; DS-W, desalinated water.

To determine whether BDSW regulates mitochondrial biogenesis, we investigated the gene expression ratio of COX Ι and RPLP0 representing mitochondrial DNA (mtDNA) and nuclear DNA (nDNA), respectively in C_2_C_12_ myotubes. BDSW increased mitochondrial biogenesis in a dose- and time-dependent manner. The expression of PGC-1α, a primary regulator for mitochondrial biogenesis, was increased in a dose-dependent manner after 3 days of BDSW treatment (Fig 1A). To determine how BDSW stimulates the increase in mitochondrial biogenesis, we next investigated the mRNA expression of various genes related to mitochondrial transcription, fatty acid oxidation, and oxidative phosphorylation. As shown in Fig 1B, BDSW increased the gene expression of transcription co-activators such as PGC-1α, mtTFA, and NRF1 and in a dose-dependent manner. To further investigate the regulatory mechanism related to mitochondrial biogenesis, we investigated gene expression related to fatty acid oxidation, oxidative phosphorylation, and mitochondrial dynamics. BDSW increased the gene expression of carnitine palmitoyltransferase 1-α (CPT1-α) and medium-chain acyl-CoA dehydrogenase (MCAD) for fatty acid oxidation, cytochrome c (CytC) for oxidative phosphorylation (Fig 2A), translocase of outer mitochondrial membrane 40 (TOMM40) and translocase of inner mitochondrial membrane 44 (TIMM44) for protein import, dynamin-related protein 1 (DRP1) for fission (Fig 2B), optic atrophy 1 (OPA1), mitofusin-1 (MFN1), and mitofusin-2 (MFN2) for fusion (Fig 2C) in a dose-dependent manner. These results suggest that BDSW is an effective stimulator of mitochondrial biogenesis.

### BDSW enhances mitochondrial enzyme activity by increasing AMPK and SIRT1 phosphorylation and PGC-1α deacetylation

**Fig 3:**
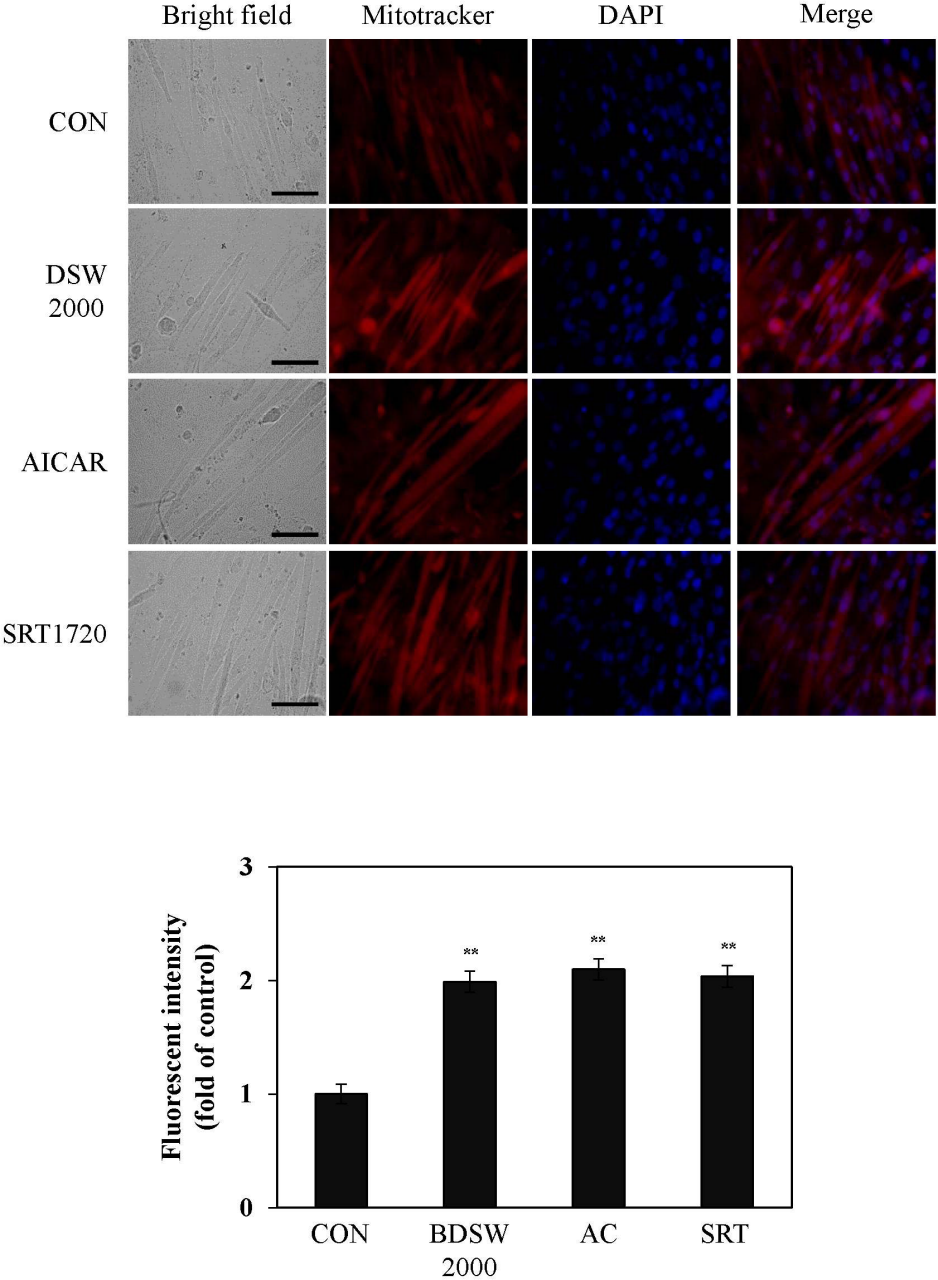
Effect of BDSW on mitochondrial mass in C_2_C_12_ myotubes. A. Representative MitoTracker Red CMXRos and DAPI staining of the C_**2**_C_**12**_ myotubes are shown at × 400 magnification. Scale bar, 50 μm. B. Quantification of MitoTracker Red CMXRos staining (n = 10 per group). Each value represents the mean ± SE of three independent experiments. **P < 0.01: significant difference vs. CON group. CON group: non-treated control group.

**Fig 4:**
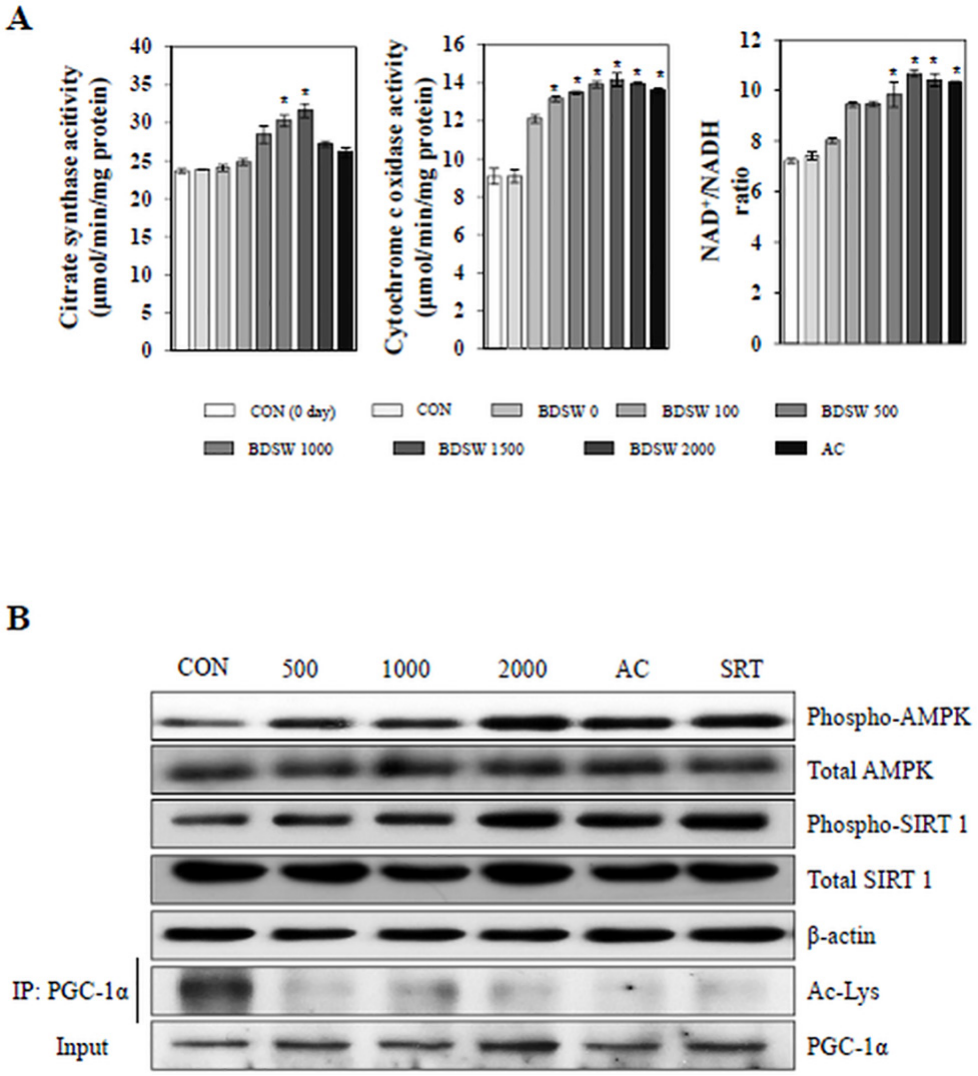
Effect of BDSW on the activity of citrate synthase and cytochrome c oxidase, the ratio of NAD^+^/NADH, and the activity of AMPK, SIRT1, and PGC-1α. A. The activity of citrate synthase and cytochrome c oxidase, and the ratio of NAD^+^/NADH were determined in cell lysates (10 μg). B. Phospho-AMPK, total AMPK, phospho-SIRT1, and total SIRT1 expression were determined in lysates (20 μg) of C_2_C_12_ myotubes after BDSW treatment for 1 h. β-actin was used as a loading control. For the activity of PGC-1α, after BDSW treatment for 6 h, PGC-1α was immunoprecipitated from nuclear extracts and then immunoblotted with either an antiacetylated lysine antibody to determine the extent of PGC-1α acetylation (Ac-Lys) or PGC-1α antibody to determine the total amount of PGC-1α. Each value represents the mean ± SE of three independent experiments. *P < 0.05: significant difference vs. CON group. CON group: DMEM media treated control group.

To examine whether BDSW-induced mitochondrial gene expression was associated with mitochondrial function, we next stained mitochondria in C_2_C_12_ cells using MitoTracker Red CMXRos. After BDSW treatment for 24 h, there was increased mitochondrial staining compared with untreated controls. As compared with AICAR, an activator of AMPK, and SRT1720, an activator of SIRT1, treated cells, the stimulatory effect of BDSW was similar to that of activators for mitochondrial biogenesis (Fig 3). To confirm that the increased mitochondrial membrane potential was associated with increased mitochondrial function, we investigated the effects of BDSW on CS activity, COX activity, and the ratio of NAD^+^ to NADH. BDSW increased CS, COX activity as well as the ratio of NAD^+^ to NADH in a dose- dependent manner (Fig 4A). Moreover, BDSW increased the phosphorylation of AMPK and SIRT1, and the deacetylation of PGC-1α, which are key signaling molecules in mitochondrial biogenesis and function (Fig 4B). These results suggest that BDSW may increase mitochondrial function via the induction of gene expression and the activation of signaling molecules.

### BDSW stimulates mitochondrial biogenesis dependent on AMPK and SIRT1 signaling pathways

**Fig 5:**
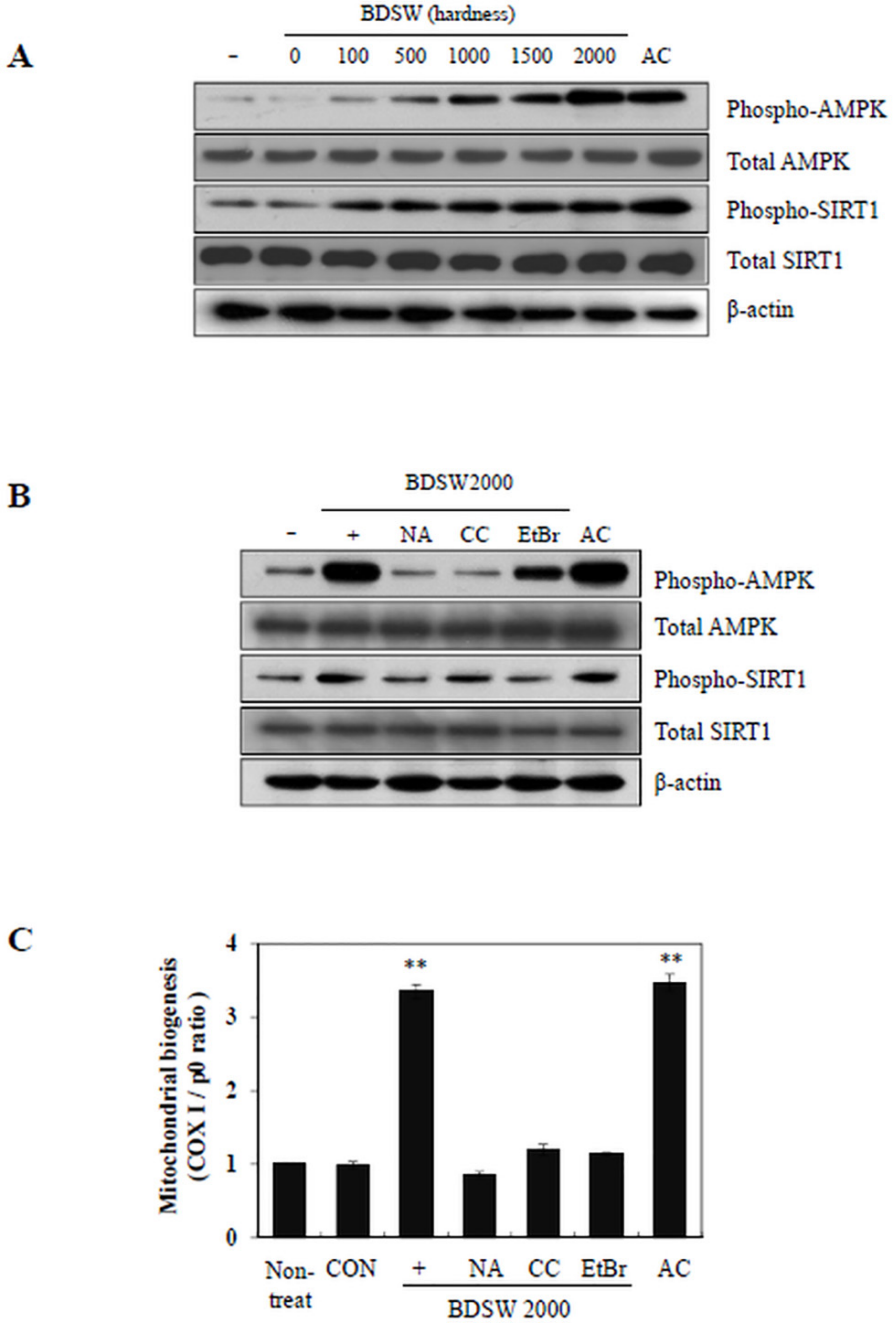
Effect of BDSW on the phosphorylation of AMPK and SIRT1. A. Western blot analyses of phospho-AMPK, total AMPK, phospho-SIRT1, and total SIRT1 in C_**2**_C_**12**_ myotubes treated with BDSW at different levels of hardness for 1 h. β-actin was used as a loading control. B. Western blot analysis following treatment with 2 mM nicotinamide (NA), 10 μM compound C (CC), and 20 ng/ml EtBr, for 1 h, followed by incubation with BDSW 2000 ppm hardness for 1 h C. For mitochondrial biogenesis using several inhibitors, after C_**2**_C_**12**_ myotubes were incubated with BDSW at hardness 2000 only, BDSW 2000 ppm with inhibitors and 1 mM AICAR (AC) for 1 day, mitochondria biogenesis were determined by the ratio of relative amount of mitochondrial DNA (COX I) and nuclear DNA (p0) quantified by real-time PCR. Non-treated group was used as the 0 day group. Each value represents the mean ± SE of three independent experiments. **P < 0.01: significant difference vs. non-treated group. CON: 1 day control group.

To confirm the mechanism by which BDSW affects mitochondrial biogenesis and function, we analyzed the phosphorylation of AMPK and SIRT1. BDSW increased the phosphorylation of AMPK and SIRT1 (Fig 5A). To further investigate the more detailed mechanisms of BDSW-mediated mitochondrial biogenesis, we next examined the effects of several inhibitors, namely, nicotinamide (NA, an SIRT1 inhibitor), compound C (CC, an ATP-competitive inhibitor of AMPK), and EtBr (an inhibitor of mtDNA synthesis). The promotion in protein phosphorylation (Fig 5B) and mitochondrial biogenesis (Fig 5C) by BDSW was inhibited by treatment with NA, CC, or EtBr. Therefore, these results suggest that the BDSW-mediated increase in mitochondrial biogenesis and function is dependent on the AMPK and SIRT1 signaling pathways.

### Drinking BDSW enhances mitochondrial biogenesis in muscles of HFD-induced obese mice

**Fig 6:**
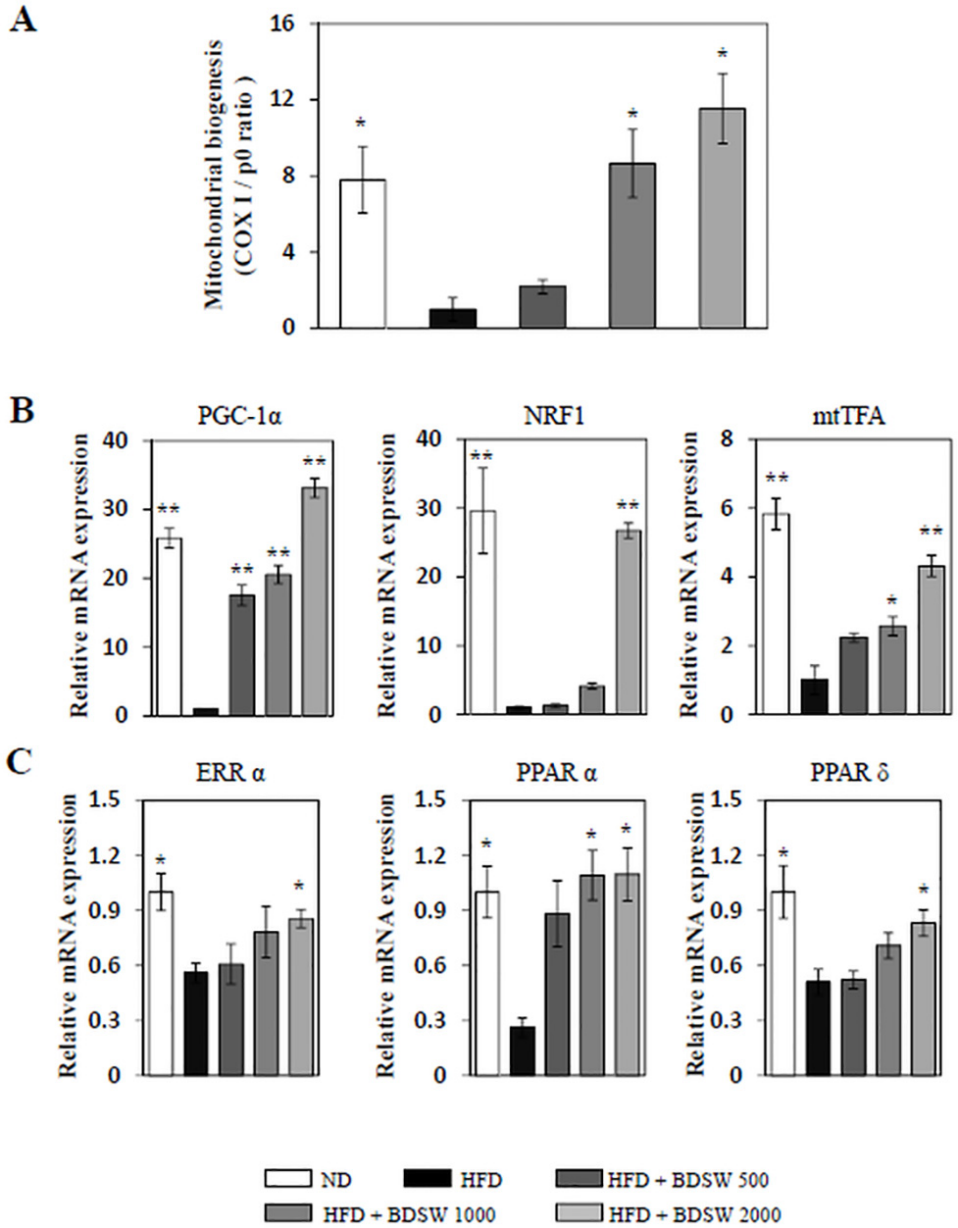
Effect of BDSW on mtDNA (A) and gene expression of PGC-1α, NRF1, and mtTFA (B), and ERRα, PPARα, and PPARδ (C) in muscles of mice fed the ND, HFD, and HFD with BDSW for 20 weeks. Each value represents the mean ± SE (n = 8 per group). *P < 0.05, **P < 0.01: significant difference vs. HFD group. ND, normal diet; HFD, high-fat diet.

To determine whether BDSW regulates mitochondrial biogenesis in vivo, we investigated mtDNA content and the expression of genes related to mitochondrial biogenesis such as PGC-1α, NRF1, and mtTFA in muscles isolated during a previous study using HFD-induced obese mice. The mitochondrial biogenesis in HFD-induced obese mice was very low; however, BDSW significantly increased mtDNA content in the muscles of HFD-induced obese mice (Fig 6A). Moreover, BDSW enhanced the expression of PGC-1α, NRF1, and mtTFA (Fig 6B) as well as estrogen-related receptor α (ERRα), peroxisome proliferator-activated receptor α (PPARα), and PPARδ, which are co-transcription factors involved in the regulation of muscle mitochondrial oxidative phosphorylation activity (Fig 6C). These results suggest that BDSW is a powerful enhancer of mitochondrial biogenesis and function in vivo.

## Discussion

Identifying the mechanisms of mitochondrial dysfunction, and developing drugs or nutrients that target the mitochondria, are well known strategies for preventing and treating mitochondria-related disease including type 2 diabetes and obesity [17]. In the present study, we demonstrated that BDSW enhances mitochondrial biogenesis and function by inducing gene expression of PGC-1α, NRF1, and mtTFA, increasing the activity of mitochondrial enzymes such as CS, CytC oxidase, and the ratio of NAD^+^ to NADH, and regulating the activity of signaling molecules such as PGC-1α, AMPK, and SIRT1 in C_2_C_12_ myotubes. Moreover, BDSW improved mitochondrial biogenesis by inducing gene expression of main transcription factors in muscles of HFD-induced obese mice. Although most of our conclusions are based on in vivo models and in vitro pharmacological interventions, the novel association between mitochondria metabolism and the anti-obesity and anti-diabetic effects of BDSW, led us to propose that the regulation of mitochondrial metabolism by BDSW is important in our understanding of obesity and diabetes. Therefore, these findings suggest that there is an association between mitochondrial metabolism and BDSW-induced inhibitory effects on obesity and diabetes.

AMP-activated protein kinase (AMPK) and the silent information regulator T1 (SIRT1), an NAD^+^-dependent deacetylase, are energy sensing molecules regulated by the AMP/ATP or NAD^+^/NADH ratio, respectively. Recent studies showed that they have common effects on diverse physiological processes such as cellular energy metabolism and inflammation as well as mitochondrial biogenesis and function [18, 19]. These similarities occur because AMPK and SIRT1 have a common mechanism that regulates the functional activity of targets such as PGC-1α, endothelial nitric oxide synthase (eNOS), and forkhead box O (FOXO). In particular, both AMPK and SIRT1 act in cooperation with PGC-1α, a key regulator of mitochondrial biogenesis, to regulate energy homeostasis in response to environmental, nutritional, and hormonal stimuli [20]. The activation of AMPK, SIRT1, and PGC-1α by BDSW may converge to enhance mitochondrial function, as demonstrated by significant increases in CS and COX activity, and induction of key metabolic genes including CPT1-α, MCAD, and CytC. Therefore, enhanced mitochondrial biogenesis and function due to BDSW, with increased activity of energy sensing molecules further support our hypothesis that BDSW improves HFD-induced obesity and type 2 diabetes by regulating energy metabolism.

Mitochondrial alterations, including reduced biogenesis and function as well as impaired morphology and dynamics, are closely associated with the development of metabolic diseases such as diabetes and obesity [21]. Mitochondrial biogenesis is the process of formation of new mitochondria, which is defined as the growth and division of pre-existing mitochondria influenced by environmental stimuli such as exercise, caloric restriction, low temperature, oxidative stress, cell division, renewal, and differentiation. It is well known that mitochondrial biogenesis is accompanied by changes in the number, size, and mass of mitochondria [22]. Mitochondrial function can have diverse effects on whole body metabolism. In particular, muscles [23] and adipose [24]tissues, which are metabolically flexible tissues, are most affected. As predicted, our findings show that BDSW enhances mitochondrial biogenesis and function with increased expression of mitochondrial genes and enzyme activity. Moreover, BDSW increased gene expression related to mitochondrial morphology and dynamics, which form an essential axis of mitochondrial quality control. Therefore, BDSW may regulate bioenergetics efficiency and energy expenditure by enhancing mitochondrial biogenesis and function.

Metabolic flexibility is the capacity of a system to adapt the oxidation of fuels, such as glucose and fatty acids, according to nutrient and oxidative availability. Conversely, metabolic inflexibility is impaired metabolic flexibility observed in peripheral tissue, such as muscle and adipose tissue, that is closelyassociated with diabetes and obesity [25]. The relationship between metabolic inflexibility and insulin resistance was observed in obese adults [26] and obese adolescents [27]. Several mitochondrial parameters including number, structure, and function, are impaired in insulin-resistant and insulin sensitive subjects [28]. These suggest that mitochondrial dysfunction might contribute to metabolic inflexibility and insulin resistance [29]. Mitochondrial deficiency in skeletal muscle is also associated with insulin resistance [30]. Taken together, our studies suggest that BDSW might enhance metabolic flexibility by enhancing mitochondrial biogenesis and function.

Optimal metabolic function of mitochondria requires the availability of essential vitamins, minerals, and other metabolites. These micronutrients are the primary cofactors that regulate basic mitochondrial function, including ATP synthesis, heme synthesis, assembly of electron transport complexes, and oxygen detoxification [31]. Deficiency of micronutrients leads to a decrease in several enzymatic activities of the electron transport complexes, while increasing the production of reactive oxidants and the functional decay of the mitochondria [32]. Recent studies showed that deficiency of essential minerals including Mg [33], Ca [34], and K [35], as well as trace minerals including Se [36], Zn [37], and V [38], was closely associated with development, prevention, and treatment of several chronic diseases including Alzheimer’s disease, stroke, hypertension, cardiovascular disease, and type 2 diabetes [39]. Although the exact roles of these minerals in metabolic disease are unknown, their deficiency results in reproductive problems, skeletal abnormalities, and diabetic cardiovascular complications. Therefore, further studies are needed to investigate the use of DSW mineral extracts in the treatment of obese and type 2 diabetes.

Mg is present as a complex with ATP in the mitochondria and is essential for the structural function of several mitochondrial proteins, including mitochondrial electron transport chain complex subunits, methylenetetrahydrofolate dehydrogenase 2, and pyruvate dehydrogenase phosphatase [40]. Studies on Mg-deficient cultured human cells [41] and animals [42] showed that mitochondrial dysfunction may induce a decrease in antioxidant defenses. However, the underlying mechanism remains unclear. Ca is a key regulator of mitochondrial function and acts as a cofactor in several mitochondrial enzymes. It plays a critical role in allosteric activation of mitochondrial enzymes such as pyruvate dehydrogenase, isocitrate dehydrogenase, and α-ketoglutarate dehydrogenase, as well as stimulation of the ATP synthase (complex V), α-glycerophosphate dehydrogenase, and adenine nucleotide translocase (ANT) [43]. The increase in cytosolic Ca level leads to increase in the levels of mitochondria-encoded COX I and mtTFA in human granulosa cells [44], as well as PGC-1α expression and mitochondrial biogenesis in rat epitrochlearis muscle [45]. On the other hand, Mendelev et al. showed that supplementation of Se increases the phosphorylation of Akt, an upstream regulator of PGC-1α, and cAMP response element-binding (CREB). In addition, Se improves mitochondrial function, including mitochondrial respiration and the activities of mitochondrial respiratory complexes I, II+III, and IV in murine hippocampal neuronal HT22 cells [46]. Moreover, it is known that low-dose supplementation of Se increases the level of glutathione (GSH) and the activities of glutathione peroxidases (GPXs), thioredoxin reductases (TRXRs), and iodothyronine deiodinases [47], as well as regulating diverse signaling pathways, including the mitogen-activated protein kinase (MAPK) [48], phosphatidylinositol 3-kinase (PI3K)-AKT [49], and NF-kB [50] pathways. Consequentially, these results support that the stimulatory effects of BDSW on mitochondrial biogenesis and function may be due to the physiological activities of BDSW components, including Mg, Ca, and Se.

For now, we speculate that the stimulatory effect of BDSW on mitochondria-associated gene expression and enzyme activity affects the synthesis of ATP or NAD+, which are known to be substrates of AMPK and SIRT1, respectively, contributing to an increase in AMPK and SIRT1 activity. As shown by Nathan et al. [51], SIRT1 plays an essential role in resveratrol-induced stimulation of AMPK and improvement of mitochondrial function, both in vitro and in vivo. We also speculate that SIRT1 activity is required for AMPK activation as well as the stimulatory effects of BDSW on mitochondrial biogenesis and function, although our present findings indicate only some indirect evidence for the association between SIRT1 and AMPK in the regulatory mechanism of BDSW on mitochondrial function. As shown in Fig 5B, NA completely inhibited the phosphorylation of both AMPK and Sirt1. In contrast, CC completely inhibited the phosphorylation of AMPK but not Sirt1. Therefore, further studies are needed to investigate what is essential for the stimulatory effect of BDSW on AMPK and SIRT1 activity as well as mitochondrial biogenesis and function, and clarify the regulatory mechanism by BDSW on AMPK and SIRT1 activity. In conclusion, our findings suggest that BDSW effectively stimulates mitochondrial biogenesis and function, and may provide a possible therapeutic approach for preventing or treating diabetes and obesity.

## Supporting Information

S1 Table Mineral content of original DSW and balanced DSW used in this study (Ex. Hardness 4680). (PDF)

S2 Table Primers for quantitative RT-PCR analysis. (PDF)
