# Supplementary material for: Stimulatory Effects of Balanced Deep Sea Water on Mitochondrial Biogenesis and Function
Source: PLoS One. 2015 Jun 12;10(6):e0129972. doi: 10.1371/journal.pone.0129972 (PMC4466323; doi:10.1371/journal.pone.0129972)
Supplement: S1 Table — (PDF) [file pone.0129972.s001.pdf]

Table 1. Mineral content of original DSW and balanced DSW used in this study (Ex. Hardness 4680)

Mineral	Original DSW (mg/L)	Balanced DSW (mg/L)
Ca	417	314
Mg	1,299	950
K	388	2.1
Na	10,794	250
Cl	18,607	665
SO^4	2,624	132
Se	4.3	0.0194
V	2.0	0.0064
Zn	8.9	N.D

N.D: not determined
