# Supplementary material for: Stimulatory Effects of Balanced Deep Sea Water on Mitochondrial Biogenesis and Function
Source: PLoS One. 2015 Jun 12;10(6):e0129972. doi: 10.1371/journal.pone.0129972 (PMC4466323; doi:10.1371/journal.pone.0129972)
Supplement: S2 Table — (PDF) [file pone.0129972.s002.pdf]

Table 2. Primers for quantitative RT-PCR analysis

	**Forward**	**Reverse**
**P^0**	GCACTTTCGCTTTCTGGAGGGTGT	TGACTTGGTTGCTTTGGCGGGATT
**COX1**	TCTACATTCGGAGCCTGAG	CTACTGATGCTCCTGCATGG
**PGC-1α**	GGAACTGCAGGCCTAACTCC	TTGGAGCTGTTTTCTGGTGC
**mtTFA**	CAGCCAGGTCCAGCTCACTA	ATTAGGAGGGTCTCGCTCCA
**NRF1**	CTCCAAACCCAACCCTGTGT	TGGTGGCCTGAGTTTGTGTT
**CPT-1α**	ACCTGAGGCATCTATTGACAG	ATGACATACTCCCACAGATGGC
**MCAD**	GGCAAATGCCTGTGATTCTT	CCATTGCGATCTTGAAACCT
**CytC**	TCCATCAGGGTATCCTCTCC	GGAGGCAAGCATAAGACTGG
**Tomm40**	AGGAGGGCACTGTCATGTCT	CTCAAACTCCACACCCACCT
**Timm44**	CCAAAGGCGAGGTGTATGAT	CTAGCCTCGAGCTGCCTAGA
**DRP1**	CTGACGCTTGTGGATTTACC	CCCTTCCCATCAATACATCC
**OPA1**	CAGCTGGCAGAAGATCTCAAG	TATGAGCAGGATTTTGACACA
**Mfn1**	AGCCCAACATCTTCATTCTGAA	CTTACAACCTTGAGCTCTTCTACCA
**Mfn2**	CATCAGTTACACCGGCTCTAACT	GAGCCTCGACTTTCTTGTTCA
**ERRα**	CACAGCCTCAGCATCTTCAA	ACTGCCACTGCAGGATGAG
**PPARα**	TTGAAGGAGCTTTGGGAAGA	AGGAAGCCGTTCTGTGACAT
**PPARδ**	ACTGGCTGTCAGGGTGGTTG	AATGCGCTGGAGCTCGATGAC
**β-actin**	AGCCATGTACGTAGCCATCC	CTCTCAGCTGTGGTGGTGAA
